# The role of matrix metalloproteinases in patients with pulmonary hypertension: data from a prospective study

**DOI:** 10.1186/s12872-021-02424-5

**Published:** 2021-12-20

**Authors:** Adriana Marc, Calin Pop, Adela-Viviana Sitar-Taut, Liviuta Budisan, Ioana Berindan-Neagoe, Dana Pop

**Affiliations:** 1 Iuliu Haţieganu” University of Medicine and Pharmacy, 400012 Cluj-Napoca, Romania; 2 Department of Cardiology, Emergency County Hospital Baia Mare, 430031 Baia Mare, Romania; 3 Faculty of Medicine Arad, West Vasile Goldis University, 310025 Arad, Romania; 4 Clinical Rehabilitaton Hospital, Cardiology, 400437 Cluj-Napoca, Romania; 5 Internal Medicine Department, 4Th Medical Clinic “Iuliu Haţieganu” University of Medicine and Pharmacy, 400012 Cluj-Napoca, Romania; 6 Research Center for Functional Genomics, Biomedicine and Translational Medicine, Iuliu – Hatieganu University of Medicine and Pharmacy, 400337 Cluj-Napoca, Romania

**Keywords:** Biomarkers, Pulmonary hypertension, Right heart failure, Matrix metalloproteinase 2, Tissue inhibitor of matrix metalloproteinase 4, N-terminal pro-B-Type Natriuretic peptide

## Abstract

**Background:**

Despite several therapies, pulmonary hypertension (PH) is still a severe disease which can lead to right heart failure. Matrix metalloproteinases (MMPs) and tissue inhibitors of metalloproteinases (TIMPs) are involved in cardiac and vascular remodeling in PH. Therefore, these biomarkers play an important role in PH patients. This study investigated whether TIMP-4, MMP-2, and N-terminal Pro-B-Type Natriuretic Peptide (NT-proBNP) plasma levels are useful in assessing the severity of PH and other clinical or echocardiographic parameters.

**Methods:**

The concentrations of MMP-2, TIMP-4, and NT-proBNP in 68 PH patients were compared with those of 12 controls without PH. All patients underwent a physical examination, echocardiography, and were checked for the presence of cardiovascular risk factors; also, plasma concentrations of MMP-2, TIMP-4, NT-proBNP, total cholesterol, and triglycerides were determined.

**Results:**

In PH patients, significantly elevated plasma levels of TIMP-4 (PH: 2877.99 ± 1363.78 pg/ml, control: 2028.38 ± 762.67 pg/ml, *p* = 0.0068) and NT-proBNP ( PH: 2405.00 pg/ml—5423.47 ± 6703.38 pg/ml, control: 411.0000 pg/ml—421.75 ± 315.37 pg/ml, *p* = 0.01) were detected. We also observed that MMP-2 and NT-proBNP were significantly increased in patients with higher WHO functional class (*p* = 0.001 for MMP-2, *p* = 0.008 for NT-proBNP), higher pressure in the pulmonary artery (*p* = 0.002 for MMP-2, *p* = 0.001 for NT-proBNP), and more severe tricuspid regurgitation (*p* = 0.001 for MMP-2, *p* = 0.009 for NT-proBNP). TIMP-4 was elevated in patients with more severe pressure in the pulmonary artery (*p* = 0.006).

**Conclusions:**

The plasma levels of TIMP-4 and NT-proBNP are higher in PH patients. MMP-2 and NT-proBNP correlates with different PH parameters severity (WHO functional class, sPAP severity, TV regurgitation severity). Therefore, plasmatic levels of MMP-2 and NT-proBNP at this kind of patients reflect disease severity and may have a prognostic role. MMP-2 can help assess the beneficial effects of PH pharmacotherapy on tissue remodeling. These remodeling biomarkers may not have a diagnostic value but they have the potential to predict survival. Nevertheless, a greater understanding of the involvement of MMPs in PH is mandatory to further explore the prognostic role and the possibilities of therapeutic MMP inhibition in PH.

## Background

Right ventricle (RV) dysfunction, defined as changes in the structure or function of the RV, is associated with poor clinical evolution regardless of the underlying disease mechanism. Right heart failure (RHF) frequently results from a gradual increase in RV afterload, caused by PH, which is commonly due to left heart failure. Chronic volume overload due to right heart diseases such as Tricuspid Valve (TV) Regugitation can also lead to RHF [1].

Pulmonary Hypertension (PH) is not a disease per se but rather a pathophysiological parameter defined by increased pressure in the pulmonary artery (with over 25 mmHg at rest) [2]. It occurs in various clinical situations and is associated with a wide range of pathophysiological changes [3]. Further, it is classified into five major groups based on their similar clinical presentation, pathological findings, hemodynamic characteristics, and treatment management [4]. PH is a severe disease in advances stages and can lead to right heart failure [5].

Several studies have found that pulmonary endothelial cell dysfunction is key to not only initiation but also progression of PH. The advanced PH stages involve the formation of intimal and plexiform lesions, endothelial apoptosis, medial and adventitial thickening of pulmonary arteries, and increased extracellular matrix (ECM) turnover, with the accumulation of ECM proteins. The ECM offers mechanical structure, elasticity, and compressibility to the vessels [6–8].

ECM Turnover is controlled by a balance between some proteolytic enzymes, such as MMPs and serine elastase, and their endogenous inhibitors [9]. The MMPs’ activity is regulated by tissue inhibitors of metalloproteinases (TIMPs) and membrane-type MMPs (MT-MMPs) [10]. Some studies have shown that MMPs and TIMPs are involved in cardiac and vascular remodelling in PH [2]. The expression of MMP2 and MT-1-MMP was detected in endothelial cells and proliferating myofibroblasts from the pulmonary arteries in the lungs of PH patients [11]. Other studies have shown that, in idiopathic PH, both in situ and in vitro, the pulmonary arteries reveal an imbalance between matrix metalloproteinases and TMP-1 and an increase in active MMP-2, leading to extracellular matrix accumulation. MMP-2 was increased in patients with idiopathic PH and may also be involved in other processes such as smooth muscle cell migration and proliferation [12, 13]. Tiede et al. have shown that plasma levels of MMP2 and TIMP2 in patients with PH correlate with disease severity and are prognostic factors. Although not diagnostic, elevated plasma levels are associated with an increased risk in these patients [14]. Schuman et al. discovered that patients with PH have increased MMP2 and TIMP4 plasma levels and that higher TIMP4 levels correlate with higher NYHA functional class and higher right ventricular hypertrophy [15].

This study aims is to investigate whether MMP2 and TIMP4 plasma concentrations correlate with disease severity, functional class (WHO), and ultrasound parameters and whether they play a prognostic role in patients with PH. These biomarkers could help monitor patients with PH and right heart failure and quantify different therapies’ effects.

## Materials and methods

### Study design

Eligible’ patients were those older than 18 years who were admitted to the Cardiology Department between January 2019 and December 2020 for dyspnea with the suspicion of PH. PH was classified according to the current guidelines and the diagnosis was set per the 2015 guidelines of the European Society of Cardiology [4]. Patients whose PH was measured echocardiographically, with systolic PAP (sPAP) over 40 mmHg, were included in the PH group of this study, and those with sPAP < 25 mmHg were included in the control group. We focused on the patients who belonged to these PH groups: PH due to left heart disease— PHLHD (we included patients who had the ejection fraction of left ventricle by more then 40%), PH due to lung diseases and/or hypoxia (PHCOPD), and chronic thromboembolic PH (CTEPH). We excluded patients with severe valvular heart disease or left heart inflow and outflow obstruction and congenital cardiomyopathies**,** sleep apnea**,** alveolar hypoventilation disorders**,** and developmental abnormalities of the lung. We also excluded group1 and group 5 PH patients according to the currernt guidelines [4]. We checked patients’ clinical history, conducted physical examination, and determined cardiovascular risk factors, echocardiography, plasma concentrations of MMP-2, TIMP-4, and NT-proBNP, total cholesterol, and triglycerides. Plasma was collected and centrifuged at 4000 rotation/min, for 12 min; samples were then frozen at -80 ºC prior to analysis. All patients were informed about the study protocol, after which they gave their, informed consent. The study was approved by the Ethics Committee of Emergency County Hospital Baia MARE, Romania (decision number 3085) as well as by the Ethics Committee of ‘Iuliu Hatieganu’ University of Medicine and Pharmacy (decision number 306).

### Analysis of biomarkers

We determined the plasma concentration of MMP-2 (Human Elisa Kit, Thermo Fisher Scientific) and TIMP-4 (Human Elisa Kit, Thermo Fisher Scientific) per the recommendations of the manufacturer. We needed 40% dilution in order for TIMP-4 to fit the kit range.

The NT-pro-BNP levels were determined at the admission to the hospital using a Roche Cardiac Reader point-of-care instrument, which calculates the levels of NT-pro-BNP from venous blood. The results ranged between 60 and 30,000 pg/ml.

### Echocardiography

We performed transthoracic 2D and colour Doppler echocardiography using the echocardiography device with a 2.5-MHz transducer on an ESAOTE MyLabClass C machine. The patients were advised to lie in the left lateral semi-recumbent or supine position during the test, per the criteria of the European Association of Echocardiography [16]. We measured right atrial and ventricular diameters, as well as sPAP, using the echo-Doppler estimation of tricuspid regurgitant wave velocity.

### Statistical packages

MedCalc version 10.3.0.0 (MedCalc Software, Ostend, Belgium) and SPSS for Windows (v16.0, IBM Corporation, Armonk, NY, USA) were used for statistical analysis. Numerical data were checked for normal distribution. Correlation between plasma biomarker concentrations and functional or morphological parameters was evaluated using Pearson and Spearman coefficients. The results of categorical variables were presented as numbers, and percentages were compared using the χ2 test. Student and Mann–Whitney U tests were used for numerical data, calculating the mean, standard deviation, and median. ANOVA or Kruskal–Wallis test were used for evaluation of the groups’ differences. A value of *p* < 0.05 was considered significant; *p* < 0.001 was considered highly significant.

## Results

The patients’ general characteristics are summarized in Table 1.

**Table 1:** Baseline characteristics of the studied population

			Global	PH	Control	*p*
			80	68	12	
Age	Mean ± SD		72.56 ± 8.1471	73.01 ± 8.56	70.00 ± 4.59	*p* = 0.0847
Gender	Nr (%)	Male	25 (31.2%)	20 (29.41)	5 (41.66)	*p* = 0.6124
		Female	55 (68.7%)	48 (70.58)	7 (58.33)	
Body mass index	Mean ± SD		29.81 ± 6.21	29.65** ± **6.2807	30.70 ± 5.98	*p* = 0.5942
WHO	Nr (%)	I	5 (6.2%)	0 (0)	5 (41.66)	*p* < 0.0001
		II [PHLHD(12), CTEPH(1), PHCOPD(1)]	21 (26.2%)	14 (20.58)	7 (58.33)	
		III [PHLHD(27), CTEPH(4), PHCOPD(6)]	37 (46.2%)	37 (54.41)	0 (0)	
		IV [PHLHD(9), CTEPH(5), PHCOPD(3)	17 (21.2%)	17 (25)	0 (0)	
TIMP-4(pg/ml)	Mean ± SD		2723.88 ± 1352.40	2846.62 ± 1399.70	2028.38 ± 762.67	*p* = 0.0068
MMP-2(ng/ml)	Mean ± SD		96.12 ± 14.49	96.76 ± 14.10	92.47 ± 16.76	*p* = 0.3479
NT-proBNP *(pg/ml)	Median (mean ± SD)		2217.50 (4673.21 ± 6430.68)	2405.00 (5423.47 ± 6703.38)	411.0000 (421.75 ± 315.37)	*p* < 0.0001
Total cholesterol (mg/dl)	Mean ± SD		159.26 ± 48.99	155.03 ± 48.66	182.91 ± 45.75	*p* = 0.0691
Triglycerides * (mg/dl)	Median (mean ± SD)		103.50 (115.35 ± 54.36)	103.00 (109.39 ± 46.84)	116.00 (149.08 ± 79.95)	*p* = 0.0659
RA size (cm^2^)	Mean ± SD		25.86 ± 10.70	26.91 ± 11.15	19.90 ± 4.51	*p* = 0.0006
LA size (cm^2^) *	Median (mean ± SD)		30.70 (35.92 ± 29.37)	31.50 (38.06 ± 31.42)	24.10 (24.15 ± 5.36)	*p* = 0.0007
RD base diameter (cm)	Mean ± SD		48.36 ± 7.89	49.31** ± **7.58	43.00** ± **7.75	*p* = 0.0097
TAPSE (mm)	Mean ± SD		20.93** ± **4.44	20.39 ± 4.36	24.00 ± 3.71	*p* = 0.0088
sPAP (mmHg)	Mean ± SD		57.50** ± **21.48	62.90 ± 18.19	26.94 ± 10.01	*p* < 0.0001

*Does not respect the normal distribution

TIMP-4: Tissue inhibitor of matrix metalloproteinase’s; MMP-2: matrix metalloproteinase; NT-proBNP: N-terminal B-type natriuretic peptide; PHLHD: pulmonary hypertension due to left heart disease; CTEPH: chronic thromboembolic pulmonary hypertension; PHCOPD: pulmonary hypertension due to lung diseases and/or hypoxia; RA: right atrium; LA: left atrium; RD: right ventricle; TAPSE: tricuspid annular plane systolic excursion; sPAP: systolic pulmonary arterial pressure

A total of 80 patients were enrolled, 68 with PH, 48 with PHLHD, 10 with CTEPH, 10 with PHCOPD, and 12 controls. The age of patients with PH was comparable to that of the control group (see Table 1).

We included 55 women (68.7%) and 25 men (31.2%). Regarding the metabolic profile, in the PH group, the total cholesterol level had a median of 155.03 ± 48.66 mg/dl and, in the control group, 182.01 ± 45.75 mg/dl (*p* = 0.065). The level of triglycerides was 109.39 ± 46.84 mg/dl in the PH group and 149.08 ± 79.95 mg/dl (*p* = 0.06) in the control group.

We calculated several echocardiographic parameters such as the right atrium area, left atrium area, right ventricle size, tricuspid annular plane systolic excursion (TAPSE), and systolic pulmonary arterial pressure (sPAP). We observed significant differences between the groups in terms of the right atrium area (26.91 ± 11.15 cm^2^ in PH group vs 19.9 ± 4.51 cm^2^ in the control group, *p* = 0.0006) and left atrium area **(**38.06 ± 31.42 cm^2^ in PH group vs 24.15 ± 5.36 cm^2^ in the control group, *p* = 0.0007). The base diameter of the right ventricle also differed between the two groups (49.31 ± 7.58 mm in PH group vs 43.00 ± 7.75 mm in the control group, *p* = 0.009); TAPSE was also measured and was 20.39 ± 4.36 mm in PH group vs 24.00 ± 3.71 mm in the control group with a significant *p* = 0.008.

### Plasma TIMP-4 concentrations

**Fig. 1:**
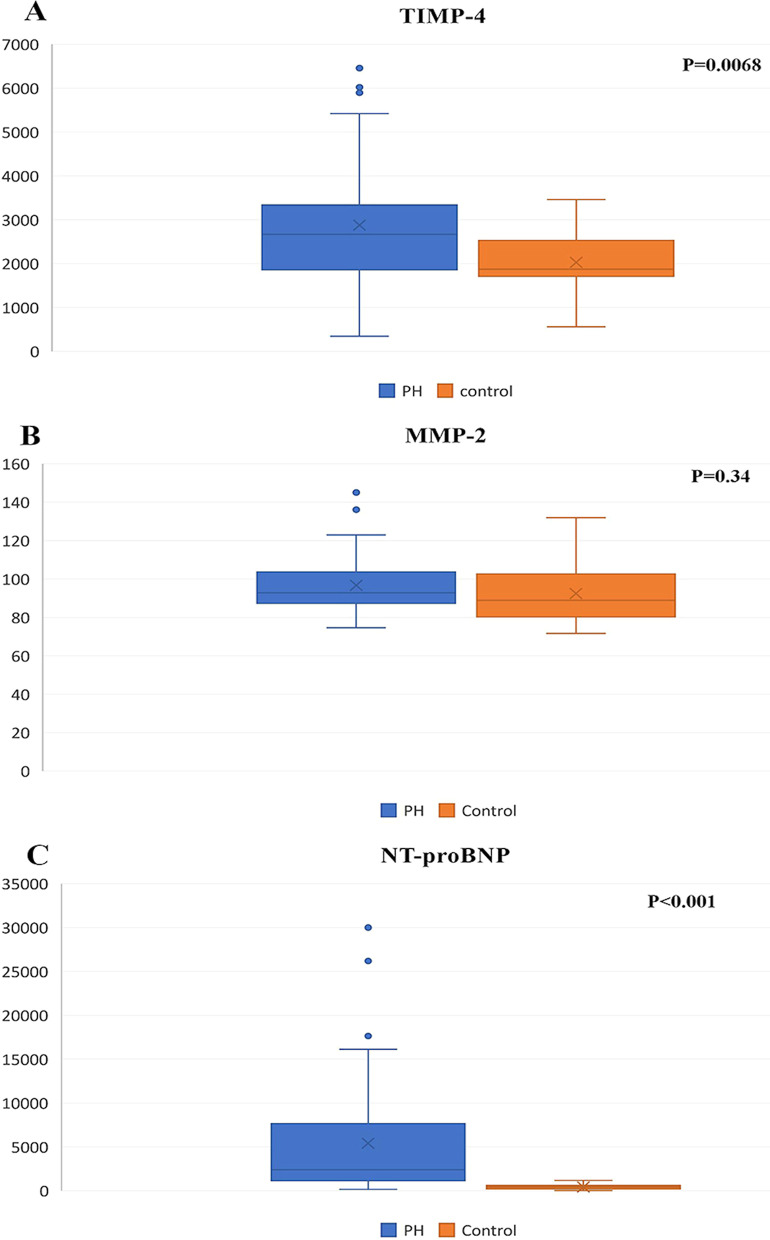
Comparison of plasma biomarker levels. Box plots show plasma biomarker concentrations in pulmonary hypertension (PH) patients (n = 68) and controls (n = 12). **a** Tissue inhibitor of matrix metalloproteinase’s (TIMP-4), **b** Matrix metalloproteinase (MMP-2), and **c** N-terminal B-type natriuretic peptide (NT-proBNP)

**Fig. 2:**
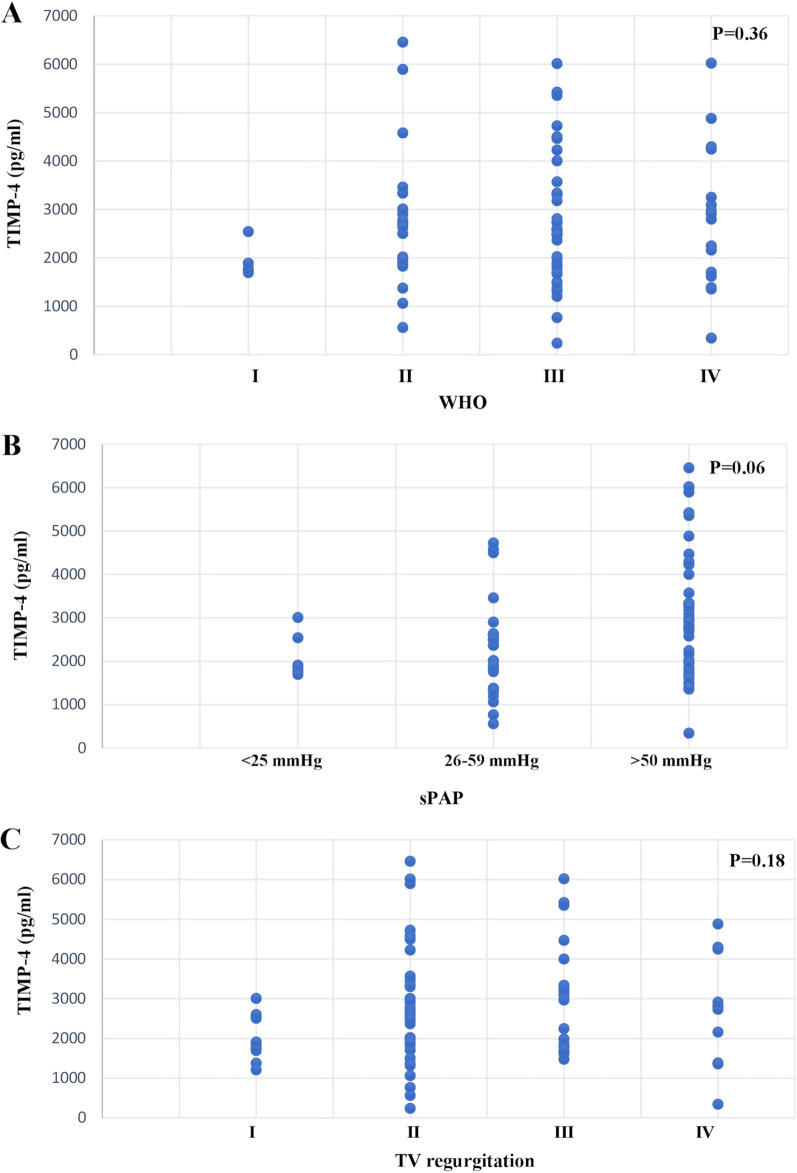
Correlation between disease severity and TIMP-4 concentration. We have box plots showing biomarker levels in different patient subpopulations. Patients were grouped based on disease severity determined as **a** WHO classification, **b** systolic arterial pressure (sPAP), and **c** the severity of TV regurgitation

**Fig. 3:**
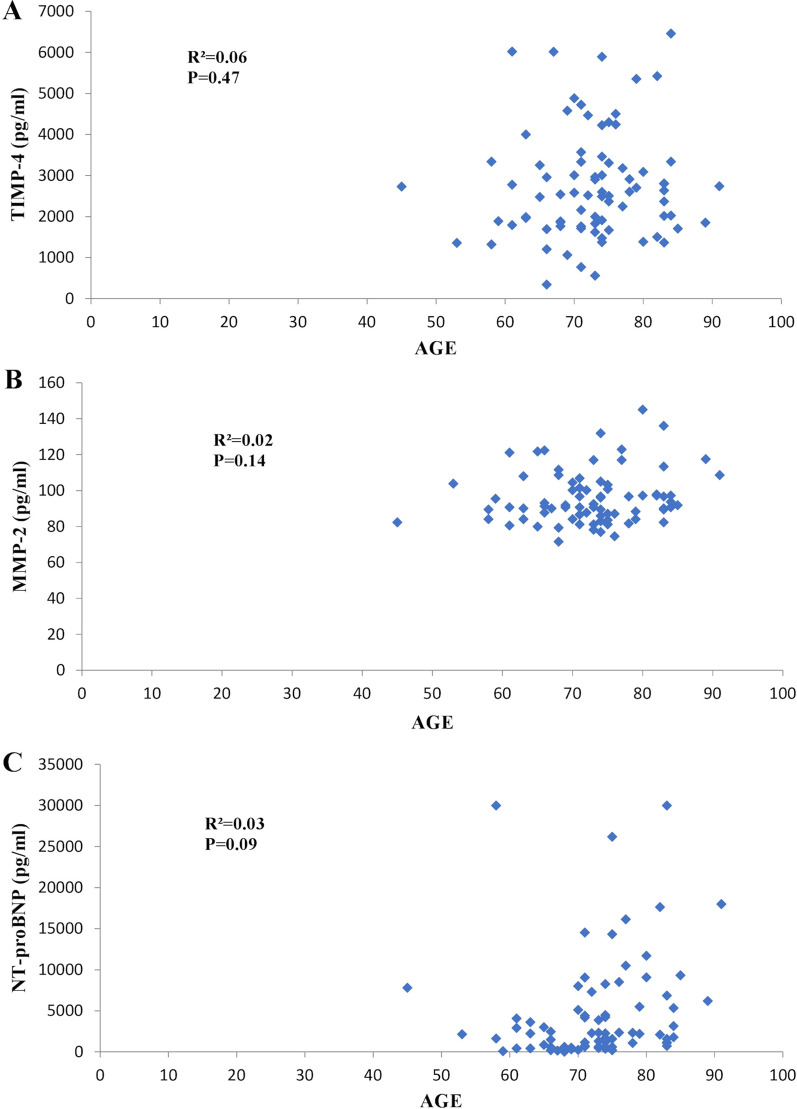
Linear regression analyses. No relationship between TIMP-4, MMP-2, NT-proBNP, and age has been found. **a** Tissue inhibitor of matrix metalloproteinases (TIMP-4), **b** Matrix metalloproteinase (MMP-2), and **c** N-terminal B-type natriuretic peptide (NT-proBNP)

Plasma TIMP-4 levels were highly increased in PH patients compared to the control group (Fig. 1a) (PH: 2877.99 ± 1363.78 pg/ml, control: 2028.38 ± 762.67 pg/ml, *p* = 0.0068). Plasma levels of TIMP-4 were different between patients with higher WHO classification but without a significant difference between the functional classes (Fig. 2a). On the other hand, in terms of the sPAP value, the TIMP-4 level was significantly different between groups, depending on the sPAP value (*p* = 0.006) (Fig. 2b). We also followed the TIMP-4 levels related to the TV regurgitation, with no significant differences detected (*p* = 0.18) (Fig. 2c). Plasma TIMP-4 concentration was not dependent on gender, age (Fig. 3a), or sex (global median value ± SD men 2438.98 ± 1399.26 vs women 2853.38 ± 1323.22 pg/ml, *p* = 0.2184).

### Plasma MMP-2 concentration

**Fig. 4:**
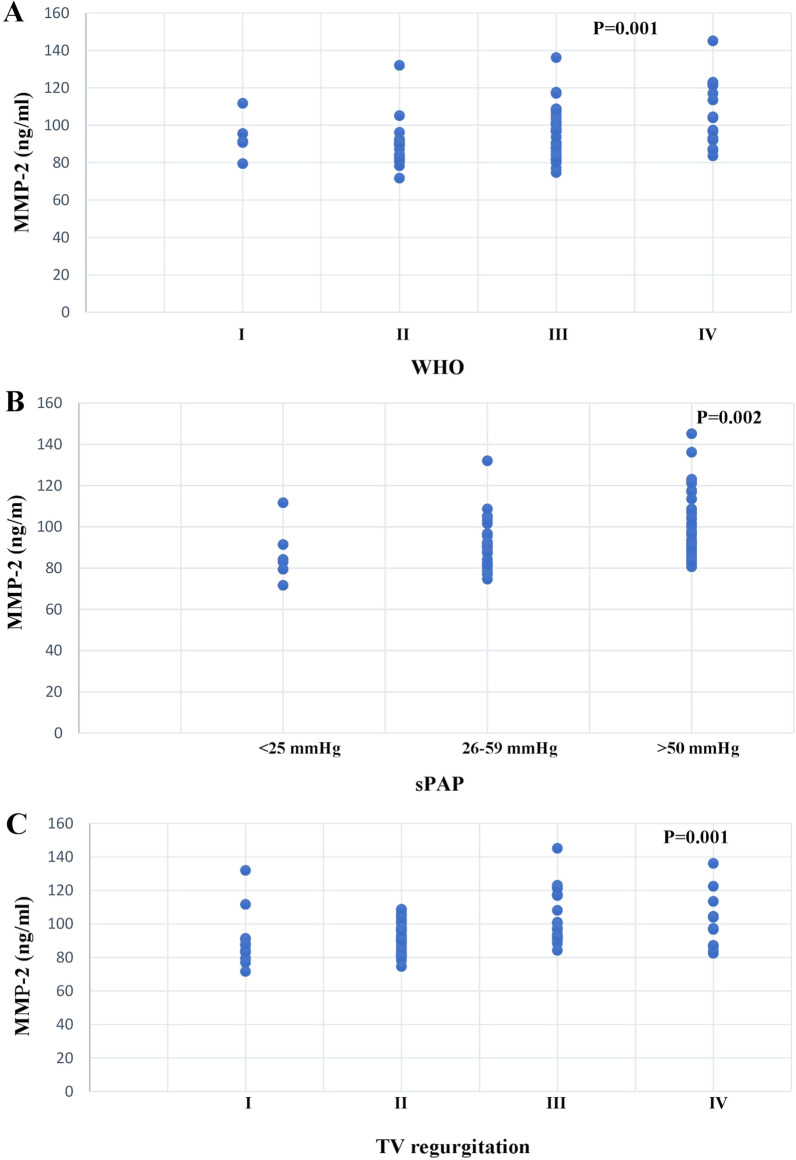
Correlation between disease severity and MMP-2 concentration. We have box plots that show biomarker levels in different patient subpopulations. Patients were grouped based on disease severity determined as **a** WHO classification, **b** systolic arterial pressure (sPAP), and **c** the severity of TV regurgitation

Plasma MMP-2 concentrations were increased in the PH group compared to the control (96.76 ± 14.10 ng/ml vs 92.47 ± 16.76) but without significant differences (Fig. 1b) (*p* = 0.34). The MMP-2 level was highly significantly increased in patients according to WHO functional class (Fig. 4a) (*p* = 0.001). MMP-2 concentrations were increased in patients with highly elevated sPAP (Fig. 4b) (*p* = 0.002) and highly significantly increased proportional to the severity of TV regurgitation (Fig. 4c) (*p* = 0.001). Plasma MMP-2 concentrations were not dependent on gender, age (Fig. 3b), or sex (global median value ± SD men 97.44 ± 13.76 vs women 95.52 ± 14.89 ng/ml, *p* = 0.5751).

### Plasma NT-proBNP concentration

**Fig. 5:**
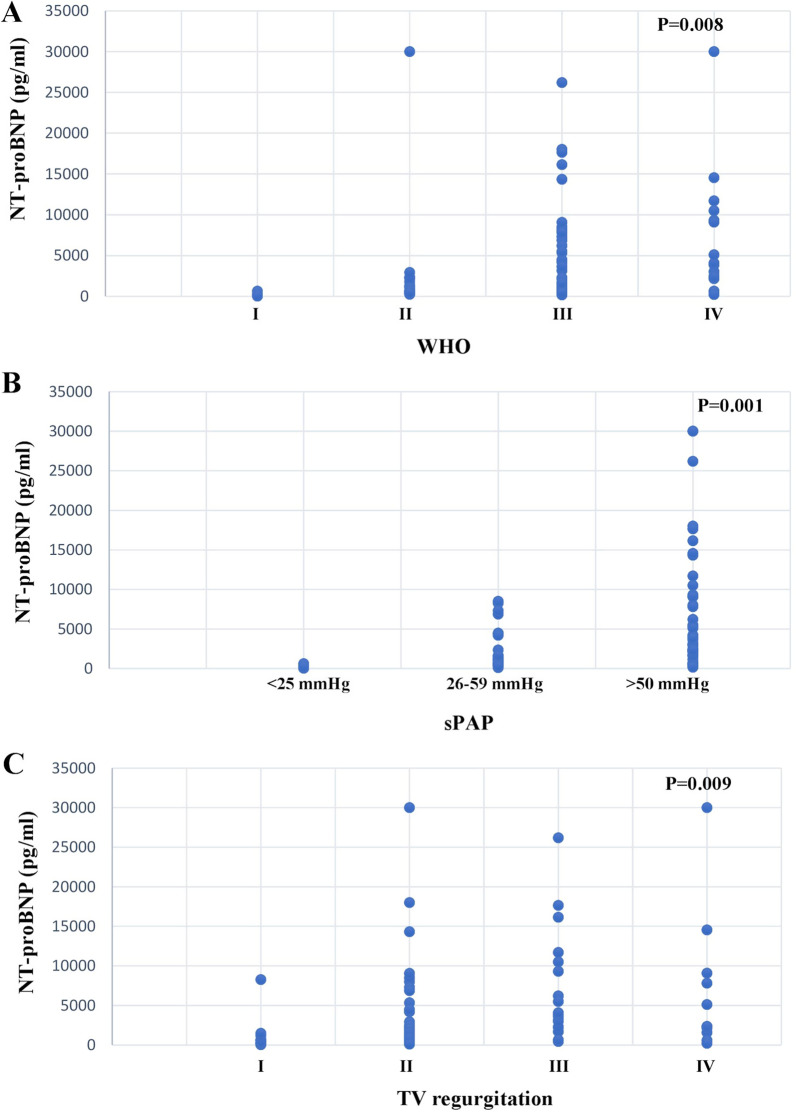
Correlation between disease severity and NT-proBNP concentration. We have box plots showing biomarker levels in different patient subpopulations. Patients were grouped based on disease severity determined by **a** WHO classification, **b** systolic arterial pressure (sPAP), and **c** the severity of TV regurgitation

We also measured the level of this marker of heart failure. Plasma levels of NT-proBNP were significantly higher in PH group compared to the control 2405.00 (5423.47 ± 6703.38) pg/ml vs 411.0000 (421.75 ± 315.37) pg/ml) (Fig. 1c) (*p* = 0.01). Further, the NT-proBNP level was highly significantly increased in patients according to the WHO functional class (Fig. 5a) (*p* = 0.008). NT-proBNP concentrations were significantly increased in patients with highly elevated sPAP (Fig. 5b) (*p* = 0.001) and significantly increased proportional to the severity of TV regurgitation (Fig. 5c) (*p* = 0.009). Plasma NT-proBNP concentrations were not dependent on gender, age (Fig. 3c), or sex (global median value ± SD men 1585 ± 7160.637 vs women 2344 ± 6139.716 ng/ml, *p* = 0.893).

We found no relationship between TIMP-4 (*p* = 0.47), MMP-2 (*p* = 0.14), and age. The NT-proBNP level tended to be age-dependent but did not reach statistical significance (*p* = 0.08, Fig. 3c).

## Discussion

Structural remodelling of the pulmonary vessels and cardiopulmonary tissue is controlled mostly by the balance of MMP-TIMP. In the areas of vascular remodelling, increased expression and activation of MMPs were observed [17].

We could not obtain organ specificity, but several studies showed an increased level of MMP-2 in PH patients. We referred to a meta-analysis with several studies that highlighted their role in PH patients [2]. Some studies showed an increased MMP-2 activity in lung tissue in patients with idiopathic PAH (IPAH); increased levels of proMMP-2 in the urine of patients with associated PAH; higher plasma concentrations of MMP-2 in PH patients with SVR < 1440 dyn·s·cm^−5^; activation of MMP-2 in patients with plexogenic pulmonary arteriopathy.

There are also studies with experimental models of PH that showed an elevated MMP-2 expression in monocrotaline-induced PH in rats; increased levels of MMP-2 in lungs in monocrotaline-treated group; increased expression of MMP-2/TIMP-2 ratio in the lungs of monocrotaline PH rats; monocrotaline-induced PH lungs with increased MMP-2 and MMP-9 activity and tumor necrosis factor expression.

Previous studies conducted on rats have shown that the plasma levels of MMP-2 and TIMP-1 reflect heart failure associated with haemodynamic, functional, and morphological changes [18]. Schumann et al. found that plasma concentrations of TIMP-4 and MMP-2 are elevated in different groups of PH (IPAH, CTEPH, and APAH-associated pulmonary arterial hypertension) patients compared to the healthy control individuals [15]. In our group, the MMP-2 level was not significantly increased in the PH group compared to the control, but, when we followed the levels of MMP-2 between milder and more severe forms of PH patients, we found MMP-2 levels to be significantly different between patients assigned as WHO I/II/III/IV and related to TV regurgitation severity. The plasma levels of MMP-2 were significantly increased in patients with highly elevated sPAP. We did not find any correlation between age or gender and the plasma MMP-2 levels.

Even organ specificity it can’t be done, TIMP-4, a cardiac inhibitor of metalloproteinases, was found to be expressed mostly in the heart, and much lower expression was detected in the kidney, pancreas, and colon. Green et al. also found that TIMP-4 may function as an acute response to tissue remodelling [19]. In our group, the TIMP-4 plasma level is significantly increased in PH patients and correlates with the sPAP value. We did not observe other significant correlations between other parameters of PH severity. Schumann et al. found that TIMP-4 levels are significantly increased in PH patients (IPAH, APAH, and CTEPH) and correlate with disease severity (different parameters such as RV hypertrophy and elevated sPAP) [15]. However, we lack knowledge about the TIMP-4 level in secondary PH patients. We found that plasma TIMP-4 concentration was neither gender nor age-dependent.

The NT-proBNP effects in heart failure patients are well-known. BNP is released at the ventricular and atrial levels as a response to the stretching of myocytes and pressure overload [20, 21]. The BNP levels are elevated in patients with valvular disease [22]. Some studies show that NTproBNP plasma level is increased in mitral regurgitation [23]. In mitral regurgitation, the BNP level correlates with mortality and the onset of chronic heart failure irrespective of the severity of mitral regurgitation on echocardiogram [24]. The level of NT-proBNP correlates with symptoms and echocardiographic severity of mitral stenosis [25]. The NT-proBNP levels are elevated in patients with COPD but not as much as in chronic heart failure [26]. In our group, the NT-proBNP level is significantly increased in PH patients and highly correlates with the sPAP value. The NT-proBNP levels are significantly different between patients assigned as WHO I/II/III/IV and between TV regurgitation severity. Once again, we confirmed the role of this important biomarker widely used in left ventricular heart failure that can also be used in PH patients.

Overall, we found that the level of TIMP-4 is elevated in PH patients proportional to the sPAP value. Although the MMP-2 level in this study was not significantly higher in patients with PH, its plasma level was a marker of PH severity, considering several parameters (WHO functional class, the sPAP level, and the severity of TV regurgitation). The expression and activity of MMP-2 have been also described as increase in idiopathic PH and it could provide data about the status of tissue remodelling or for monitoring the response to treatment with different target molecules for PH treatment: e.g. administration of the bosentan (dual endothelin receptor agonist) attenuated the monocrotaline-induced up regulation of MMP-2, TIMP-1 and endothelial NO synthase expression. [12, 27]. Also, everolimus or alagebrium in combination with sildenafil demonstrate supplementary regulatory effects on MMPs 2 and 9, as well as functional responses on pulmonary artery pressure [28]. Several vasoselective dihydropyridine calcium channel blocker have been shown to improve PH via regulation of MMP/TIMPs: lercanidipine, showed improvement in PAH subjects by reducing serum MMP-9 levels with no modification of proMMP-2 activity or TIMP-1 level; amlodipine, administered to the monocrotaline induced PH rats was shown to inhibit MMP-2 activity, platelet activation, and plexogenic proliferation [29, 30]. Different studies showed that MMP-2 and TIMP-1 levels in PH patients reflect disease severity and could predict outcome. They are not being of diagnostic value, but elevated levels are significantly associated with increased risk in patients with PH [14, 31]. Therefore, monitoring plasma levels of MMP-2 and TIMP-4 could bring complementary data, which can be added to NT-proBNP utilization. The NT-proBNP role in heart failure is well-known. We know that its level increases in valvulopathies [22, 24, 25] and in the presence of lung diseases, especially COPD [29, 32, 33] and in pulmonary thromboembolism [34, 35] but also with the degree of right ventricular dysfunction [36]. In this study, we found that NT-proBNP level statistically significantly differs among different patient subpopulations: in terms of functional WHO class, sPAP level, and the severity of TV regurgitation, confirming its prognostic and severity role in this PH category and right heart failure. However, these biomarkers need reassessment before being used prognostically or guiding specific treatment.

Our study has several potential limitations. We used a relatively small and heterogeneous group of patients with different causes of PH, and no analysis was undertaken concerning PH etiology. We determined the biomarker levels only at a single time point, and they might need longitudinal evaluation and correlation with medical management. Important limitations concerning the diagnostic of PH that was made in our study by echocardiography. In the current guidelines, the right heart catheterization (RHC) is mandatory in confirming PH diagnosis for groups 1 and 4 (CTEPH) of PH patients [4]. Group 1 (especially IPAH) patients were excluded from our study as they had good anamnesis and clinical correlations. For group 2 of PH (PHLHD), the indication for RHC is IIb and, for group 3 of PH (PHCOPD), RHC is not recommended (class III) unless therapeutic consequences are to be expected. Also, there are new comparative data—echocardiography versus RHC, which showed that hemodynamic changes found in transthoracic echocardiography, like sPAP, can be estimated with acceptable precision given an adequate TRV envelope [37, 38]. These are the reasons why we considered only the echocardiographic data instead of invasive RHC.

## Conclusions

We found that plasma levels of TIMP-4 and NT-proBNP are higher in PH patients. MMP-2 and NT-proBNP correlates with different PH parameters severity (WHO functional class, sPAP severity, TV regurgitation severity). Therefore, plasmatic levels of MMP-2 and NT-proBNP at this kind of patients reflect disease severity and they have a prognostic role. MMP-2 can help assess the beneficial effects of PH pharmacotherapy on tissue remodeling.

These remodeling biomarkers may not have a diagnostic value but may have the potential to predict survival. Nevertheless, a greater understanding of the involvement of MMPs in PH is mandatory to further explore the prognostic role and the possibilities of therapeutic MMP inhibition in PH.

## Data Availability

All data generated or analysed during this study are included in this published article. The data are available from the corresponding author on reasonable request.

## Abbreviations

PH: Pulmonary hypertension
sPAP: Pressure in the pulmonary artery
MMPs: Matrix metalloproteinases
TIMPs: Tissue inhibitors of metalloproteinases
NT-proBNP: N-terminal Pro-B-Type natriuretic peptide
TV: Tricuspid valve
ECM: Extracellular matrix
PHLHD: PULMONARY hypertension due to left heart disease
CTEPH: Chronic thromboembolic pulmonary hypertension
PHCOPD: Pulmonary hypertension due to lung diseases and/or hypoxia
TAPSE: Tricuspid annular plane systolic excursion
